# Foot biomechanics in patients with advanced subtalar- and mid-tarsal joint osteoarthritis and poorly responding to conservative treatment

**DOI:** 10.1186/s13047-023-00689-x

**Published:** 2023-11-28

**Authors:** Kevin Deschamps, Karel Mercken, Pieter Verschuren, Maarten Eerdekens, Eline Vanstraelen, Sander Wuite, Giovanni A. Matricali

**Affiliations:** 1 Department of Rehabilitation Sciences, Musculoskeletal Rehabilitation Research Group, KU Leuven, Campus Brugge, Belgium; 2 Clinical Motion Analysis Laboratory, University Hospitals Leuven, Pellenberg, Belgium; 3 Faculty of Medicine, KU Leuven, Leuven, Belgium; 4 Department of Orthopedics, University Hospitals Leuven, Leuven, Belgium; 5 Institute for Orthopaedic Research & Training, University Hospitals Leuven, Leuven, Belgium; 6 Department of Development & Regeneration, KU Leuven, Leuven, Belgium

**Keywords:** Osteoarthritis, Subtalar joint, Mid-tarsal joint, Kinematics, Kinetics

## Abstract

**Background:**

A comprehensive insight into the effects of subtalar- and mid-tarsal joint osteoarthritis on lower limb’s biomechanical characteristics during walking is lacking. Our goal was to assess joint kinematics and kinetics and compensatory mechanisms in patients with subtalar and mid-tarsal joint osteoarthritis.

**Methods:**

Patients with symptomatic and radiographically confirmed osteoarthritis of the subtalar and mid-tarsal (*n* = 10) and an asymptomatic control group (*n* = 10) were compared. Foot joint kinematics and kinetics during the stance phase of walking were quantified using a four-segment foot model.

**Results:**

During pre-swing phase, the tibio-talar range of motion in the sagittal plane of the patient group decreased significantly (*P* = 0.001), whereas the tarso-metatarsal joint range of motion in the sagittal plane was greater in the pre-swing phase (*P* = 0.003). The mid-tarsal joint showed lower transverse plane range of motion in the patient group during the loading response and pre-swing phase (*P* < 0.001 resp. *P* = 0.002). The patient group showed a lower Tibio-talar joint peak plantarflexion moment (*P* = 0.004), peak plantarflexion velocity (*P* < 0.001) and peak power generation in the sagittal plane (*P* < 0.001), and a lower mid-tarsal joint peak adduction and abduction velocity (*P* < 0.001 resp. *P* < 0.001) and peak power absorption (*P* < 0.001).

**Conclusions:**

These findings suggest that patients with subtalar and mid-tarsal joint osteoarthritis adopt a cautious walking strategy potentially dictated by pain, muscle weakness, kinesiophobia and stiffness. Since this poorly responding population faces surgical intervention on the short term, we recommend careful follow-up after fusion surgery since biomechanical outcome measures associated to this post-surgical stage is lacking.

**Supplementary Information:**

The online version contains supplementary material available at 10.1186/s13047-023-00689-x.

## Introduction

Osteoarthritis (OA) of the foot leads to chronic pain, is associated with walking difficulties and has functional implications with reduced quality of life [1]. Despite a high confirmed prevalence of symptomatic and radiographic foot OA, extensive studies of OA in the subtalar (STJ) and mid-tarsal joint (MTJ) (including talonavicular and calcaneocuboid joints) remain scarce compared to knee, hip, and tibio-talar joint OA [2–4]. Following its triplanar motion, the STJ has a strong coupling function with the proximal joints [5, 6]. Distally, the STJ is coupled with the MTJ to provide an efficient tarsal locking mechanism [7].

Joint degeneration may occur at the STJ or MTJ in an isolated way, however, the involvement of both joints has been reported in various patient groups [8]. Next to distinct clinical symptoms and anatomical changes, altered foot biomechanics during static and dynamic activities have been quantified [1, 4, 9]. As such, it has been reported that the phasic activity of extrinsic foot muscles may be affected causing muscle atrophy, joint contractures and deformity in advanced stages of OA [10, 11]. Based on a literature review, Lithgow et al. concluded that patients with midfoot OA have increased loading of the heel and midfoot during walking [12]. The same authors suggested that more detailed biomechanical studies are needed to better understand midfoot OA [12]. Several studies investigated the joint kinematics in ankle and foot OA using three-dimensional gait analysis, showing mostly a reduced range of motion, reduced walking speed and even (mal)adaptive motion patterns at the contralateral non-affected foot [12–15]. The strength of these studies lies in the fact that a multi-segmented approach is used [16]. The multi-segment kinematic behavior of the foot in presence of combined STJ and MTJ osteoarthritis has, to our knowledge, not yet been addressed. Moreover, to fully understand the effect of STJ and MTJ osteoarthritis on the lower limb’s characteristics during walking, kinetic and mechanical function must be investigated as well. Eerdekens et al. was the first to investigate both multi-segment foot kinematics and kinetics in patients with tibio-talar OA [17]. They demonstrated a decrease in ankle kinetics and no change in distal foot joints kinetics, meaning that the mechanical dysfunction of the ankle is not compensated by the distal foot joints [17]. A study conducted by Deleu and co-workers quantified the effect of concomitant foot deformities on the multi-segment foot kinematics and kinetics of patients with ankle osteoarthritis [18]. They reported altered frontal and transverse plane joint angles and moments in a cohort facing a so-called cavus osteoarthritis foot and concluded that the varus inclination of the ankle joint was compensated by transverse plane alterations in the midfoot-metatarsus angle.

Investigating the kinetic behavior of the foot and ankle complex during walking encompasses several notable benefits for patients with STJ and MTJ osteoarthritis, such as individual management of conservative treatment options, general monitoring of foot function and presurgical planning. In addition, these multi-segment kinetic foot models might allow us to investigate the coping strategies of other foot joints.

The aim of this study was to develop new insights in the mechanical function of the ankle and foot joints in patients with STJ and MTJ osteoarthritis. First, we hypothesized that patients with STJ and MTJ osteoarthritis would demonstrate lower range of motion and peak power joint kinetics in the tibio-talar and mid-tarsal joint compared to a healthy group. The second hypothesis was that (mal)adaptive biomechanical patterns would occur in the tarso-metatarsal and first metatarsophalangeal (MTP1) joint. Finally, we hypothesized that the patient group will report poor to moderate physical function and quality of life.

## Methods

### Study design and study population

An observational study was carried out at the University Hospital Leuven following ethical approval of the local ethical committee and all participants provided informed consent (ML9038).

Between 2015 and 2022, 76 patients consulting our university hospital with symptomatic and radiographically confirmed arthritis of STJ and MTJ were invited to participate in the clinical motion analysis laboratory to collect a prospective database. All selected patients had a lack of satisfactory symptomatic response regarding conservative treatment, including foot orthoses, shoe modification and physiotherapy treatment (including massage, joint mobilization, strengthening, proprioceptive exercises). Since considerable heterogeneity existed within this population with respect to the medical background of the foot pathology, a further selection of patients was considered based on the following inclusion criteria: i) diagnosis of unilateral talonavicular, calcaneocuboid and subtalar osteoarthritis (at least a grade 2 Kellgren-Lawrence osteoarthritis score confirmed by two senior orthopedic surgeons) [19], ii) the ability to walk 100 m barefoot without rest or walking aids. Exclusion criteria were: i) radiographic signs of osteoarthritis in other foot joints (Kellgren OA score > 1), ii) uni- or contralateral joint fusions in the lower limb, iii) presence of systemic and or neurological diseases (e.g. rheumatoid arthritis, hemophilia, Charcot-Marie Tooth) or a history of osteoarthritis in any of the lower limb joints based on their available medical history and on clinical assessment, iv) people younger than 18 years. Finally, only 10 patients met the predetermined in- and exclusion criteria and were therefore selected for the current study.

Reference data serving as the control group in the current study was selected from an existing database of asymptomatic male adults. Selection of the specific control data was based on the age and BMI of the patient data.

### Materials and methods

#### Radiographic data

Clinical and radiographic data were extracted from the patient electronic medical record. Standard weightbearing plain radiographs of the ankle and foot were used. Imaging was performed at the first consultation and at maximum 3 months before their gait analysis. Concerning the ankle, an anteroposterior, a mediolateral orientation and a mortise view were used. Concerning the foot, an anteroposterior, a mediolateral and a ¾ internal rotation view. Two senior foot surgeons evaluated the radiographs and a consensus meeting was organized regarding the scoring of the different joints on the Kellgren-Lawrence scale.

#### Gait assessment

Gait analysis was performed in the institutional’s Clinical Movement Analysis Laboratory. Data were obtained when the participants walked along 10-m walkway, surrounded by an optoelectronic motion capture consisting of 10 T-10 cameras (100 Hz, Vicon Motion Systems Ltd., Oxford, United Kingdom). A force plate (Advanced Mechanical Technology, Watertown, MA, USA) and a superimposed pressure plate (Footscan, dimensions 0.5 m ∙ 0.4 m, 4096 sensors, 2.8 sensors/cm^2^; RSscan International, Paal, Belgium) were an integral part of the walkway. Both plates were dimensionally matched and were built into the floor. An RSscan 3D synchronization box was used to synchronize and calibrate the plantar pressure and force data.

The force and pressure plate data were sampled at 200 Hz.

Multi-segment foot kinematics and kinetics were assessed by placing retroreflective markers (Ø = 10 mm) to the participants’ feet and shanks following the marker placement protocol of the Instituto Ortopedico Rizzoli (IOR) Foot Model [20]. Subsequently, the patients were instructed to walk along the aforementioned walkway at their own pace until at least five representative trials were recorded.

Data processing incorporated manual marker labelling and definition of the individual gait cycles using Nexus software (Vicon Motion System Ltd, Oxford Metrics, Oxford, UK). Following this post-processing routine, the IOR-4segment-model-1 described by Deschamps et al. [21] was applied. This model calculates 3D intersegment joint rotations between the following adjacent segments: shank-calcaneus (Sha-Cal), the calcaneus-midfoot (Cal-Mid), midfoot-metatarsus (Mid-Met), hallux and metatarsus (Hallux); as well as the following non-adjacent segments: calcaneus and metatarsus (Cal-Met). The following terminology was used with respect to the aforementioned adjacent inter-segment angle calculations (joints): tibio-talar joint between shank and calcaneus, mid-tarsal joint between calcaneus and midfoot, tarso-metatarsal joint between midfoot and metatarsus, and the first metatarsophalangeal joint (MTP 1) between hallux and metatarsus.

Joint centers were respectively defined as the midpoint between both malleoli (tibio-talar joint), the midpoint between the navicular and cuboid bone (mid-tarsal joint), the second metatarsal base (tarso-metatarsal joint), and the projection of the MTP1 marker halfway to the floor (MTP1). Subsequently, ground reaction forces and moments (captured by the force and pressure plate) were distributed over the different segments of the IOR-4segment-model-1 using a validated proportionality scheme [22]. For every time frame of the gait cycle, the resulting pressure in each of these segments, compared with the total pressure, provided the proportion of the total ground reaction force to each corresponding segment. Then, we calculated inertial parameters based on the mass of each segment and their geometric solids. The mass of the foot was distributed at a 30/30/30/10 (rearfoot/midfoot/forefoot/hallux) percent rate. Joint kinetics were computed starting from the distal joint and progressing proximally, using Newton–Euler equations using an in-house custom inverse dynamic analysis program (ACEP-Manager, Matlab2016a, The Mathworks, Natick, US). Following data-processing, normalization of all waveforms for a full stance phase was performed.

Regarding the joint kinematics the following outcome variables were investigated: 1) range of motion (RoM), defined as the difference between the maximum and minimum value in a kinematic waveform, and calculated for three subphases of stance including loading response (0–20% stance phase), midstance and terminal stance together (21–83% stance phase) and pre-swing (84–100% stance phase) [23].

Finally, peak internal joint moment, peak dorsiflexion and plantarflexion angular velocity and peak power generation and absorption (joint moment multiplied with angular velocity) at the different joints were quantified as kinetic outcome measures.

#### Patient reported outcome measures

All patients completed the Short-Form-36 (SF-36) [24] and an adapted Foot Function Index (FFI) validated for a Dutch speaking population [25, 26], and scored their pain experience using a visual Analog Scale (VAS). The SF-36 quantified physical and mental well-being in eight health concepts. A high score indicated a good outcome. The FFI quantified foot health, pain and foot health related quality of life with a score between 0 and 100. A higher score indicates a lower functioning person in terms of pain, disability and activity restriction.

### Statistical analysis

IBM SPSS Statistics 20 (IBM Corp, Armonk, NY, USA) was used to perform the statistical analyses. Data were assessed for normality with the Shapiro–Wilk test. An independent samples t-test (if the assumptions for normality are achieved) or a Mann Whitney U test (if the assumptions for normality are not achieved) was used to compare the demographic parameters. One-way ANCOVA tests were computed to analyze group differences between the zero-dimensional parameters of the control and patient group. Walking speed was considered as a covariate since the control group had a significantly higher walking speed. To guard against inflation of type I error but maintain statistical power across the multiple comparisons made up on the multiple variables it was decided to adjust the conventional alpha level and adopt a *P*-value of < 0.01. Mean differences (with 95% confidence intervals) and effect size (ES) were also calculated. The effect size for Cohen’s d value was calculated and interpreted as follows: d = 0.20 (small effect), *d* = 0.50 (medium effect), *d* = 0.80 (large effect), and *d* = 1.30 (very large effect) [27]. Trends were still considered within the usual benchmark, α = 0.05.

The degree of kinematic coupling was evaluated by calculating the cross-correlation coefficient of the angular displacement curves of adjacent segments across the stance phase [28]. Based on previous publications [29], it was decided to analyse the coupling between four inter-segment rotations: i) Sha-Cal Inversion/Eversion with Cal-Met Dorsiflexion/Plantarflexion, 3) Sha- Cal Inversion/Eversion with Cal-Met Inversion/Eversion, ii) Sha-Cal Inversion/Eversion with Cal-Met Adduction/Abduction. The following qualitative benchmarks were used when evaluating this cross-correlation coefficient: 1) strong coupling > 0.7 or <  − 0.7), 2) moderate coupling between ( −)0.3 to ( −)0.69 and weak coupling between − 0.3 and 0.3 [30].

## Results

### Study population

Baseline demographic and clinical characteristics can be found in Table 1. The patient population had an average of age 44.3 ± 17.8 (range, 20 – 71) years with an average BMI of 26.1 ± 5.7 (range, 17.7 – 38.9) kg/m^2^. Etiology of the pathogeneses were variable, despite the stringent in- and exclusion criteria. There was a significant decrease in walking speed (*P* = 0.004) and step length (*P* = 0.001) between both groups.

**Table 1:** Demographic and clinical characteristics of the patient and control group

Variable	Patients (*n* = 10)	Controls (*n* = 10)	*P*-value	Mean difference (95% CI interval)
**Demographic variables**				
Male/Female	5/5	10/0		
Age in years	44.3 ± 17.8	37.1 ± 6.5	0.246	7.2 [-5.4, 19.8]
BMI in kg/m^2^	26.1 ± 5.7	24.6 ± 3.8	0.529 †	1.5 [-3.1, 6.1]
Side (Left/Right)	7/3	5/5		
Pathogenesis				
– primary arthritis	1			
– posttraumatic arthritis	7			
– other	2			
**Spatiotemporal variables**				
Cadence (steps/min)	102.4 ± 7.9	107.5 ± 8.9	0.197	5.1 [-2.8, 13.0]
Walking speed (m/s)	1.0 ± 0.2	1.2 ± 0.1	0.004*	0.2 [0.1, 0.3]
Step length (m)	0.6 ± 0.09	0.7 ± 0.04	0.001*	0.1 [0.04, 0.17]
Step time (s)	0.6 ± 0.05	0.6 ± 0.04	0.181	0.0 [-0.04, 0.04]
**Patient Reported Outcome Measures (PROM)**				
VAS pain score				
(10-point scale)				
– median	6.3			
– average	6.8 ± 1.3			
FFI pain subscore (36-point scale)	18.6 ± 6.4			
FFI disability subscore (36-point scale)	20.3 ± 5.7			
SF-36 form				
– Physical functioning	51.0			
– Role limitations due to physical health	40.0			
– Role limitations due to emotional problems	53.3			
– Energy/fatigue	56.0			
– Emotional well-being	54.8			
– Social functioning	62.5			
– Pain	41.8			
– General health	66.0			

All outcomes are represented as the mean ± standard deviation; *P*-values indicated with * represent significance (*p* < 0.01); all *P*-values are calculated using an independent samples t-test; *P*-values indicated with † are calculated using a Mann Whitney U-test (non-parametric statistics); *FFI* Foot Function Index; *VAS* Visual analogue Score; *SF-36* Short Form 36, *OA* osteoarthritis

Inverse dynamics calculation in the patient population could not be calculated for one symptomatic subject. Therefore, statistical analysis was performed on only nine participants for the kinetic parameters (dropout = 1). The covariate, walking speed, was significantly related to one kinematic variable and several kinetic variables (see Table 2–3 and Table S-3).

**Table 2:** Kinematic characteristics during three different phases of stance and planes for both groups of the Tibio-talar, Mid-tarsal, Tarso-metatarsal and MTP 1 joint. Summary of mean range of motion (RoM) and standard deviation (in degrees)

	Patients (*n* = 10)	Controls (*n* = 10)	Mean difference [95% CI]	*P*-value	Cohen’s D
TIBIO-TALAR JOINT					
Sagittal plane					
Loading response	4.2 ± 1.0	5.3 ± 1.6	1.1 [-0.1, 2.3]	0.013 †	0.31
Midstance & terminal stance	9.1 ± 3.8	8.7 ± 3.0	0.4 [-2.8, 3.6]	0.872	< 0.01
Pre-swing	9.5 ± 3.3	19.2 ± 3.8	9.7 [6.4, 13.0]	0.001*	0.49
Frontal plane					
Loading response	2.1 ± 1.8	2.8 ± 0.9	0.7 [-0.6, 2.0]	0.645	0.01
Midstance & terminal stance	1.8 ± 1.1	5.6 ± 1.1	3.8 [2.8, 4.8]	< 0.001*	0.67
Pre-swing phase	1.6 ± 1.5	2.7 ± 1.1	1.1 [-0.2, 2.3]	0.316	0.06
Transverse plane					
Loading response	1.6 ± 0.8	1.8 ± 0.8	0.2 [-0.5, 1.0]	0.521	0.03
Midstance & terminal stance	4.4 ± 2.0	6.0 ± 2.4	1.6 [-0.5, 3.6]	0.715	0.01
Pre-swing	1.9 ± 0.8	2.4 ± 1.1	0.5 [-0.4, 1.4]	0.775	0.01
MID-TARSAL JOINT					
Sagittal plane					
Loading response	1.8 ± 1.3	2.3 ± 0.8	0.5 [-0.6, 1.4]	0.956	< 0.01
Midstance & terminal stance	7.7 ± 3.4	5.4 ± 3.5	2.3 [-0.9, 5.6]	0.402	0.04
Pre-swing	7.1 ± 4.7	11.4 ± 3.2	4.3 [0.5, 8.1]	0.187	0.10
Frontal plane					
Loading response	2.3 ± 1.1	2.4 ± 0.7	0.1 [-0.8, 1.0]	0.961	< 0.01
Midstance & terminal stance	2.7 ± 2.0	2.0 ± 1.2	0.7 [-0.9, 2.2]	0.157	0.11
Pre-swing	1.5 ± 1.3	2.0 ± 1.3	0.5 [-0.7, 1.7]	0.514	0.03
Transverse plane					
Loading response	0.7 ± 0.3	2.5 ± 0.9	1.8 [1.2, 2.4]	< 0.001*	0.56
Midstance & terminal stance	2.2 ± 1.0	2.7 ± 1.0	0.5 [-0.4, 1.5]	0.883	< 0.01
Pre-swing	2.1 ± 1.3	5.9 ± 1.6	3.8 [2.4, 5.2]	0.002*	0.46
TARSO-METATARSAL JOINT					
Sagittal plane					
Loading response	3.1 ± 1.7	3.2 ± 2.1	0.1 [-1.7, 1.9]	0.149	0.12
Midstance & terminal stance	2.8 ± 2.0	7.5 ± 1.6	4.7 [2.9, 6.3]	0.002*	0.44
Pre-swing	6.3 ± 3.6	2.3 ± 0.9	4.0 [1.6, 6.4]	0.003*	0.41
Frontal plane					
Loading response	1.9 ± 1.5	3.2 ± 1.5	1.3 [-0.1, 2.7]	0.169	0.11
Midstance & terminal stance	2.8 ± 2.0	2.7 ± 1.1	0.1 [-1.4, 1.7]	0.510	0.03
Pre-swing	4.1 ± 3.3	2.3 ± 1.5	1.8 [-0.6, 4.2]	0.061	0.19
Transverse plane					
Loading response	2.1 ± 1.1	2.3 ± 1.2	0.2 [-0.9, 1.3]	0.911	< 0.01
Midstance & terminal stance	2.3 ± 1.2	4.2 ± 2.4	1.9 [0.04, 3.6]	0.200	0.10
Pre-swing	3.3 ± 1.9	4.5 ± 2.2	1.2 [-0.7, 3.1]	0.568	0.02
MTP1 JOINT					
Sagittal plane					
Loading response	4.6 ± 3.3	7.5 ± 2.9	2.9 [0.03, 5.8]	0.163	0.11
Midstance & terminal stance	8.7 ± 6.6	17.5 ± 4.3	8.8 [3.6, 14.0]	0.103	0.15
Pre-swing	17.7 ± 8.7	16.5 ± 4.9	1.2 [-5.5, 7.8]	0.271	0.09
CALC-MET ANGLE					
Frontal plane					
Loading response	2.5 ± 2.2	2.1 ± 1.0	0.4 [-1.2, 2.0]	0.498	0.03
Midstance & terminal stance	4.8 ± 2.8	2.6 ± 1.4	2.2 [0.1, 4.2]	0.001*	0.50
Pre-swing	5.1 ± 4.0	7.8 ± 2.6	2.7 [-0.5, 5.8]	0.976	< 0.01

*CI* confidence interval; * ANCOVA: significant *P*-values (< 0.01) are noted in bold; † ANCOVA: significant *P*-values (*p* < 0.05) for the covariate ‘walking speed’ in the analysis; MTP 1 = first metatarsophalangeal joint; CALC-MET = calcaneus-metatarsal angle

**Table 3:** Sagittal, frontal and transverse plane kinetics (mean and standard deviation) of the Tibio-talar and Mid-tarsal joint

Variable	Patients (*n* = 10)	Controls (*n* = 10)	Mean difference [95% CI]	*P*-value	Cohen’s D
**TIBIO-TALAR JOINT**					
**Sagittal plane**					
Peak PF moment (Nm/kg)	1.0 ± 0.3	1.5 ± 0.1	0.5 [0.3, 0.7]	0.004*	0.44
Peak DF velocity (°/s)	58.8 ± 13.7	86.8 ± 17.9	28.0 [12.0, 43.9]	0.036	0.26
Peak PF velocity (°/s)	108.2 ± 30.0	231.9 ± 45.7	123.7 [85.1, 162.3]	< 0.001*	0.60
Peak power generation (W/kg)	0.9 ± 0.5	2.8 ± 0.8	1.9 [1.3, 2.6]	< 0.001* †	0.57
Peak power absorption (W/kg)	0.4 ± 0.2	0.6 ± 0.3	0.2 [-0.04, 0.4]	0.480	0.03
**Frontal plane**					
Peak INV moment (Nm/kg)	0.2 ± 0.1	0.1 ± 0.1	0.1 [-0.01, 0.2]	0.199	0.11
Peak INV velocity (°/s)	23.8 ± 13.4	54.4 ± 11.5	30.6 [18.2, 43.2]	0.003*	0.26
Peak EV velocity (°/s)	34.5 ± 25.9	51.6 ± 14.7	17.1 [-4.0, 38.1]	0.430	0.04
Peak power generation (W/kg)	0.05 ± 0.04	0.05 ± 0.04	0.00 [-0.04, 0.05]	0.654	0.01
Peak power absorption (W/kg)	0.04 ± 0.01	0.22 ± 0.13	0.18 [0.09, 0.27]	0.036 †	0.26
**Transverse plane**					
Peak ADD moment (Nm/kg)	0.08 ± 0.09	0.03 ± 0.03	0.05 [-0.02, 0.12]	0.129	0.15
Peak ADD velocity (°/s)	28.3 ± 11.7	52.2 ± 18.7	23.9 [8.4, 39.5]	0.241 †	0.09
Peak ABD velocity (°/s)	33.4 ± 13.2	43.0 ± 33.6	9.6 [-15.9, 35.1]	0.676	0.01
Peak power generation (W/kg)	0.03 ± 0.03	0.03 ± 0.02	0.00 [-0.02, 0.03]	0.071	0.20
Peak power absorption (W/kg)	0.03 ± 0.03	0.13 ± 0.08	0.10 [0.04, 0.16]	0.113	0.16
**MID-TARSAL JOINT**					
**Sagittal plane**					
Peak PF moment (Nm/kg)	0.8 ± 0.2	1.1 ± 0.1	0.3 [0.1, 0.5]	0.248	0.09
Peak DF velocity (°/s)	47.0 ± 15.9	50.5 ± 14.7	3.5 [-11.7, 18.9]	0.865	< 0.01
Peak PF velocity (°/s)	77.5 ± 46.8	144.9 ± 48.8	67.4 [19.7, 115.2]	0.120	0.15
Peak power generation (W/kg)	0.6 ± 0.6	1.4 ± 0.4	0.8 [0.4, 1.3]	0.070	0.20
Peak power absorption (W/kg)	0.4 ± 0.2	0.3 ± 0.2	0.1 [-0.2, 0.3]	0.432	0.04
**Frontal plane**					
Peak INV moment (Nm/kg)	0.04 ± 0.05	0.04 ± 0.03	0.00 [-0.04, 0.04]	0.587	0.02
Peak INV velocity (°/s)	24.2 ± 13.9	24.0 ± 13.3	0.2 [-13.4, 13.7]	0.516	0.03
Peak EV velocity (°/s)	22.8 ± 9.7	35.7 ± 16.8	12.9 [-0.8, 26.6]	0.328	0.06
Peak power generation (W/kg)	0.03 ± 0.02	0.02 ± 0.03	0.01 [-0.02, 0.03]	0.947	< 0.01
Peak power absorption (W/kg)	0.04 ± 0.03	0.10 ± 0.06	0.06 [0.003, 0.11]	0.326	0.06
**Transverse plane**					
Peak ADD moment (Nm/kg)	0.014 ± 0.03	0.002 ± 0.001	0.012 [-0.01, 0.03]	0.136	0.14
Peak ADD velocity (°/s)	27.5 ± 11.2	84.8 ± 21.9	57.3 [39.9, 74.6]	< 0.001* †	0.60
Peak ABD velocity (°/s)	16.3 ± 5.8	46.7 ± 11.7	30.4 [21.1, 39.6]	< 0.001*	0.62
Peak power generation (W/kg)	0.03 ± 0.03	0.02 ± 0.01	0.01 [-0.01, 0.04]	0.042	0.25
Peak power absorption (W/kg)	0.02 ± 0.02	0.25 ± 0.1	0.23 [0.15, 0.30]	< 0.001* †	0.55

*CI* confidence interval; * ANCOVA: significant *P*-values (< 0.01) are noted in bold; † ANCOVA: significant *P*-values (*p* < 0.05) for the covariate ‘walking speed’ in the analysis; *PF* plantarflexion; *INV* inversion; *ADD* adduction; *DF* dorsiflexion; *EV* eversion; ABD = abduction

### Range of motion and coupling

**Fig. 1:**
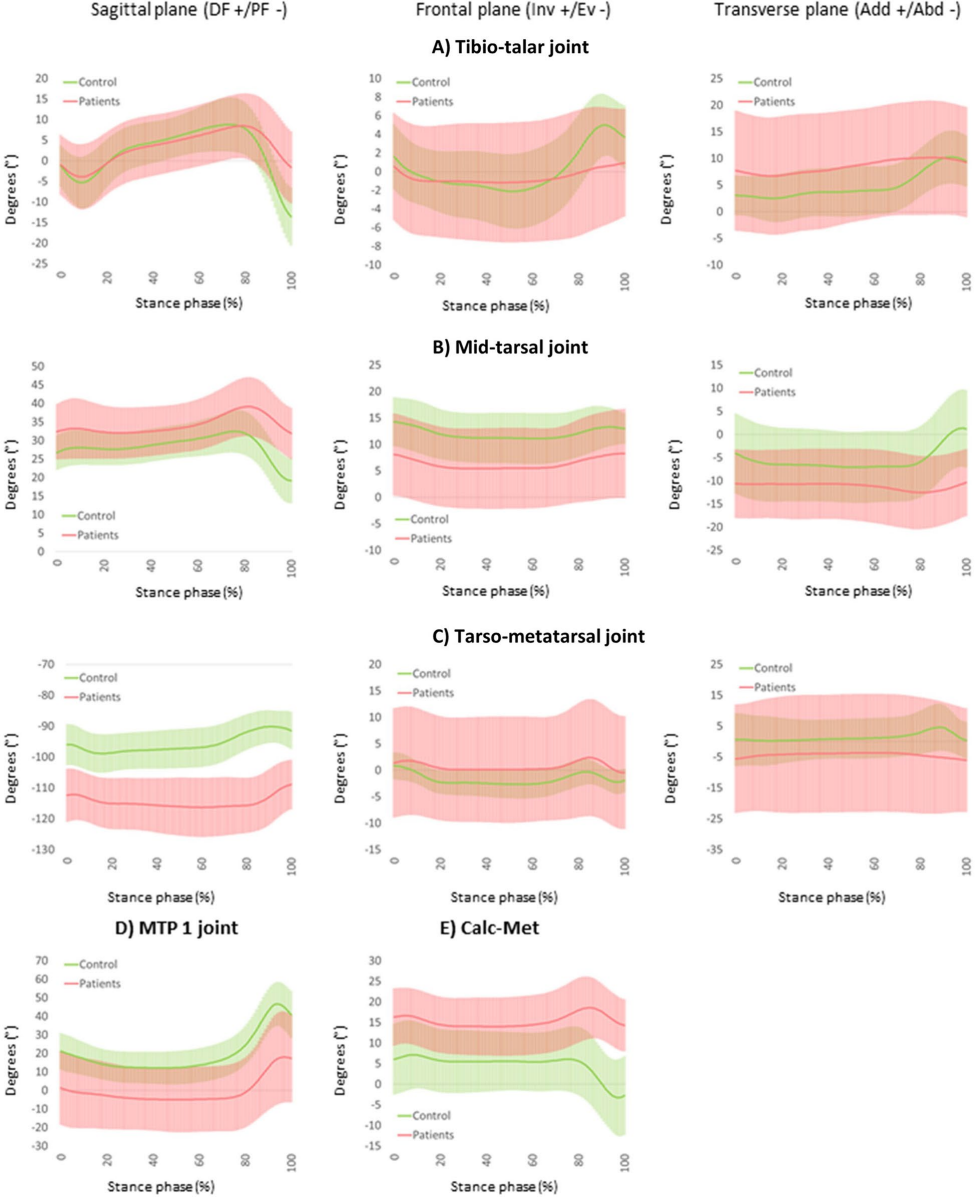
Multi-segment foot kinematics (mean and standard deviation bands) of the patient group (red) and control group (green) in the sagittal (DF + /PF-), frontal (Inv + /Eve-) and transverse plane (Add + /Abd-)

The kinematic outcome variables for the tibio-talar joint, mid-tarsal joint, tarso-metatarsal joint, and MTP 1 joint are shown in Table 2 and in Fig. 1.

#### Loading response

The mid-tarsal joint RoM of the patient group was significantly lower in comparison with the control group (mean difference 1.8°, CI 95% 1.2 to 2.4; *P* < 0.001). A trend towards lower sagittal plane RoM in the tibio-talar joint during the loading response phase was observed in the patient group (mean difference 1.1°CI, 95% -0.1 to 2.3; *P* = 0.013). No other significant differences were observed in this subphase of the walking cycle. 

#### Midstance and terminal stance

In this phase the patient group demonstrated a significantly lower RoM in the frontal plane of the tibio-talar joint (mean difference 3.8°, CI 95% 2.8 to 4.8; *P* < 0.001). In contrast, a significantly higher RoM was found in the frontal plane of the Calc-Met angle of the patient group (mean difference 2.2°, CI 95% 0.1 to 4.2; *P* = 0.001). In the tarso-metatarsal joint, a lower RoM was seen in the sagittal plane in the patient group (mean difference 4.7°, CI 95% 2.9 to 6.3; *P* = .002)).

#### Pre-swing

In the last phase of stance, the mean sagittal plane RoM in the tibio-talar joint of the patient group was significantly lower compared to the control group (mean difference 9.7°, 95% CI 6.4 to 13.0; *P* = 0.001). In contrast, the sagittal plane RoM of the tarso-metatarsal joint of the patient group was significantly higher compared to the control group (mean difference 4.0°, 95% CI 1.6 to 6.4; *P* = 0.003). The transverse plane data showed a significantly lower mobility of the mid-tarsal joint in the patient group compared to the control group (mean difference 3.8°, CI 95% 2.4 to 5.2; *P* = 0.002).

#### Coupling

A trend towards a lower of coupling between the Sha-Cal Inv/Ev movement and the Cal-Met Inv/Ev movement (patients r = 0.04, controls r = -0.5, *P* = 0.012) was found. Moreover, a trend towards lower coupling between the Sha-Cal Inv/Ev movement and the Cal-Met DF/PF was observed (patients r = -0.3, controls r = -0.7, *P* = 0.035).

### Joint kinetics

The tibio-talar joint kinetics differed significantly (*P* < 0.01) in peak plantarflexion moment (ES = 0.44), peak plantarflexion velocity (ES = 0.60), peak power generation in the sagittal plane (ES = 0.57) and peak inversion velocity in the frontal plane (ES = 0.26), being all lower in the patient group (Table 3). Trends were shown (*P* < 0.05) in peak dorsiflexion velocity (ES = 0.26) and peak power absorption in the frontal plane (ES = 0.26), representing lower values in the patient group compared with the control group (Table 3).

The mid-tarsal joint kinetic parameters have shown significant differences between means in peak adduction and abduction velocity which were lower compared to the control group (ES = 0.60 and 0.62 respectively). The peak power absorption in the transverse plane was significantly lower in the patient group (ES = 0.55) while the peak power generation showed a trend being higher in the patient group in the same plane (ES = 0.25) (Table 3).

No significant differences were observed in the tarso-metatarsal joint and MTP1 joint kinetics (Table S-3, Supplementary file).

### Foot function index and VAS for pain

**Fig. 2:**
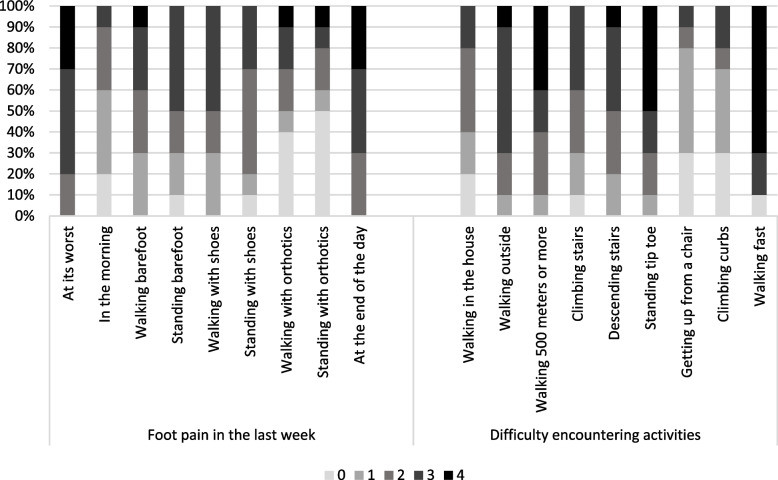
One hundred percent stacked bar chart about the subdivision of the Foot Function Index in ‘Foot pain in the last week’ and ‘Difficulty encountering activities’ in the patient group (*n* = 10). Score 0 = no pain / no effort, score 1 = some pain / some effort, score 2 = quite a bit of pain / quite a bit of effort, score 3 = a lot of pain / a lot of effort, score 4 = intolerable pain / intolerable effort

Details of the FFI score distribution are visualized in Fig. 2. The activity patients found most difficult to carry out was walking fast, followed by standing tip toe and walking 500 m or more. The median VAS score was 6.3, ranging from 5.0 to 8.7.

## Discussion

The current study includes patients with end-stage osteoarthritis at the STJ and MTJ facing failed conservative treatment and were scheduled for surgical intervention. The key findings suggests that in the patient group, both the kinematic and kinetic parameters of the involved joints are lower compared with the control group and that the distal foot joints do not present major (mal)adaptive kinematic and kinetic joint patterns. It is reasonable to assume that the reported debilitating pain, functional limitations and limited physical health may be principal drivers behind the observed foot biomechanics. 

### Joint kinematics and coupling

Even though the applied multi-segment foot model is not able to differentiate the kinematic behavior of the subtalar and tibiotalar joint separately, some significant differences were captured in the currently reported data. Evidence for a lower sagittal plane RoM was observed during loading response and pre-swing combined with less frontal plane RoM during midstance and terminal stance. The former may originate from the joint structural damage but may also represent a more cautious weight acceptance (e.g. kinesiophobia, pain-driven fear) at initial contact whereas the lower sagittal plane RoM during pre-swing may originate from weakness of the calf muscles. Another plausible explanation for the difference in pre-swing RoM may be the adoption of a pull-off strategy (passive propulsion) instead of a push-off pattern (active propulsion). The lower frontal plane tibio-talar joint RoM during midstance and terminal stance explains why this patient population reports difficulties with walking on uneven terrain and highlights probably a critical loss of foot mobile adapter function [31]. Also, the lower transversal plane RoM observed at the mid-tarsal joint contributes to this loss of foot function. In addition, it seems that the windlass mechanism is affected in the patient population as evidenced by the lower frontal plane RoM at the Cal-Met angle, the latter being a surrogate measure for the mechanism [20].

From the cross-correlation coefficients one can assume that the synchronous motion between the rearfoot and the forefoot is affected. The latter is an important finding since this may play a considerable role in the overall success rate of surgical interventions. The authors believe that restoring these coupling mechanisms is unrealistic in advanced stages of OA using the current available treatment methods, and that this should be discussed with the patient, e.g. prior to fusion surgery.

### Joint kinetics

The peak plantarflexion moment in the sagittal plane of the tibio-talar joint was significantly reduced. This could be explained by pain or stiffness at the hindfoot joints, atrophy of the calf muscles and a reduced walking speed leading to the pull-off strategy. Evidence for the adoption of the latter strategy is also provided by the lower peak plantarflexion velocity and the lower peak power generation in the tibio-talar joint.

The trend towards an increased transverse plane peak power generation at the mid-tarsal joint reflects a foot that has lost its mobile adapter function and may on the other hand originate from concomitant ankle and hindfoot malalignment. Deleu et al. reported similar observations in patients suffering from post-traumatic ankle osteoarthritis [18].

### Foot function index and VAS for pain

The FFI and the VAS score have shown that OA of the STJ and MTJ has a relevant impact on daily life, with pain progressively occurring towards the evening and most reported with speed walking and long-distance walking. It is therefore not surprising that the current findings reflect a pain-induced walking pattern. Similar findings have been reported in patients following triple arthrodesis in the hindfoot [32].

### Clinical relevance

We recommend close follow-up of these patients since they have poorly responded to conservative treatment and will therefore enter a care pathway involving foot surgery. This pathway typically comprises fusion surgery of the involved joints and it is well known that the primary goals of this surgery are to decrease pain and to improve overall function. To our knowledge, there is a lack of published research on the post-op rehabilitation of patients undergoing this surgery and it is believed that the current study provides valuable information which may guide future research in this domain. 

### Limitations

The main limitation in this study is the inability to provide measurements of independent tibiotalar and subtalar join motion since the current study used a traditional passive motion capture system with retroreflective skin-markers [33, 34]. In addition, the effects of STJ and MTJ arthrosis are difficult to differentiate from each other as combined cases were included in the study. Another limitation of our study is the difference in sex between patients and the control group. The patient group consisted of 50% women, while there were none in the control group. Research by Wunderlich et. al. showed an important difference in foot anatomy in men and women [35]. This imbalance may therefore influence the overall outcome of the study. Our study is also limited by a small patient population size. This makes the interpretation of results less representative and increases the likelihood of an overestimation of the effect size [36]. Another limitation which is worth mentioning is the 7-year age difference between groups.

Lastly, participants had to perform the walking trials throughout a barefoot condition. Therefore, the effect of footwear on the walking pattern cannot be estimated, hence, it is well-known that a shod condition is often more comfortable for these patients [37].

## Conclusion

Key findings suggest that the involved joints are characterized by a different range of motion and peak joint kinetics and that the distal foot joints do not present major (mal)adaptive patterns. We conclude that these patients adopt a pull-off strategy probably dictated by pain, muscle weakness and kinesiophobia.

### Supplementary Information

**Additional file 1:** **Table S-3.** Sagittal, frontal and transverse plane kinetics (mean and standard deviation) of the Lisfranc and first metatarsophalangeal joint.

## Data Availability

Data available on simple request.
