# Supplementary material for: Foot biomechanics in patients with advanced subtalar- and mid-tarsal joint osteoarthritis and poorly responding to conservative treatment
Source: J Foot Ankle Res. 2023 Nov 28;16:85. doi: 10.1186/s13047-023-00689-x (PMC10683126; doi:10.1186/s13047-023-00689-x)
Supplement: Supplementary file 1 — Additional file 1: Table S-3. Sagittal, frontal and transverse plane kinetics (mean and standard deviation) of the Lisfranc and first metatarsophalangeal joint. [file 13047_2023_689_MOESM1_ESM.docx]

**Table S-3.** Sagittal, frontal and transverse plane kinetics (mean and standard deviation) of the Lisfranc and first metatarsophalangeal joint.

| **Variable** | **Patients**  **(n = 10)** | **Controls**  **(n = 10)** | **Mean difference**  **[95% CI]** | ***P*-value** | **Cohen’s D** |
| --- | --- | --- | --- | --- | --- |
| **Tarso-metatarsal joint** | | | | | |
| **Sagittal plane** | | | | | |
| Peak PF moment (Nm/kg) | 0.4 ± 0.1 | 0.5 ± 0.1 | 0.1 [-0.03, 0.2] | 0.663 | 0.01 |
| Peak DF velocity (°/s) | 74.7 ± 41.5 | 58.5 ± 21.7 | 16.2 [-16.9, 49.3] | 0.071 | 0.20 |
| Peak PF velocity (°/s) | 38.7 ± 20.6 | 68.3 ± 30.8 | 29.6 [3.4, 55.7] | 0.712 | 0.01 |
| Peak power generation (W/kg) | 0.3 ± 0.3 | 0.4 ± 0.1 | 0.1 [-0.1, 0.3] | 0.687 | 0.01 |
| Peak power absorption (W/kg) | 0.04 ± 0.03 | 0.04 ± 0.04 | 0.00 [-0.03, 0.04] | 0.503 | 0.03 |
| **Frontal plane** | | | | | |
| Peak INV moment (Nm/kg) | 0.06 ± 0.08 | 0.13 ± 0.05 | 0.07 [0.003, 0.14] | 0.182 | 0.12 |
| Peak INV velocity (°/s) | 38.9 ± 25.5 | 41.4 ± 19.5 | 2.5 [-20.2, 25.1] | 0.861 | < 0.01 |
| Peak EV velocity (°/s) | 47.6 ± 29.9 | 56.2 ± 26.8 | 8.6 [-19.8, 37.0] | 0.736 | 0.01 |
| Peak power generation (W/kg) | 0.09 ± 0.14 | 0.06 ± 0.05 | 0.03 [-0.08, 0.13] | 0.111 | 0.16 |
| Peak power absorption (W/kg) | 0.02 ± 0.03 | 0.05 ± 0.04 | 0.03 [-0.01, 0.06] | 0.798 † | 0.01 |
| **Transverse plane** | | | | | |
| Peak ADD moment (Nm/kg) | 0.02 ± 0.03 | 0.03 ± 0.03 | 0.01 [-0.01, 0.04] | 0.880 | < 0.01 |
| Peak ADD velocity (°/s) | 37.7 ± 18.4 | 49.3 ± 26.4 | 11.6 [-11.1, 34.4] | 0.745 | 0.01 |
| Peak ABD velocity (°/s) | 42.5 ± 18.0 | 85.1 ± 40.5 | 42.6 [11.4, 74.0] | 0.145 | 0.14 |
| Peak power generation (W/kg) | 0.1 ± 0.1 | 0.2 ± 0.1 | 0.1 [-0.01, 0.2] | 0.604 | 0.02 |
| Peak power absorption (W/kg) | 0.02 ± 0.03 | 0.10 ± 0.09 | 0.08 [-0.01, 0.15] | 0.166 | 0.12 |
| **Metarso-phalangeal joint** | | | | | |
| **Sagittal plane** | | | | | |
| Peak PF moment (Nm/kg) | 0.10 ± 0.07 | 0.13 ± 0.06 | 0.03 [-0.04, 0.10] | 0.531 | 0.03 |
| Peak DF velocity (°/s) | 195.4 ± 96.6 | 319.9 ± 96.6 | 124.5 [28.0, 221.1] | 0.247 | 0.09 |
| Peak PF velocity (°/s) | 92.3 ± 40.9 | 224.9 ± 104.3 | 132.6 [53.4, 211.7] | 0.078 | 0.19 |
| Peak power generation (W/kg) | 0.04 ± 0.06 | 0.09 ± 0.05 | 0.05 [-0.003, 0.11] | 0.173 | 0.12 |
| Peak power absorption (W/kg) | 0.4 ± 0.3 | 0.5 ± 0.2 | 0.1 [-0.1, 0.4] | 0.891 | < 0.01 |

CI = confidence interval; * ANCOVA: significant *P*-values (<0.01) ​​are noted in bold; † ANCOVA: significant *P*-values (p<0.05) for the covariate ‘walking speed’ in the analysis; PF = plantarflexion; INV = inversion; ADD = adduction; DF = dorsiflexion; EV = eversion; ABD = abduction.
